# Debunking educational myths: towards evidence-based simulation training

**DOI:** 10.31744/einstein_journal/2025Suppl_3ED0001

**Published:** 2025-12-03

**Authors:** Dario Cecilio-Fernandes, Alessandra Mazzo, Brena Carvalho Pinto de Melo, Mariana Santos Alecrim Molina, Priscilla Cerullo Hashimoto, Joyce Kelly Barreto Silva, Desiree Gonçalves, Thomaz Bittencourt Couto

**Affiliations:** 1 Erasmus MC University Medical Centre Rotterdam Institute of Medical Education Research Rotterdam Rotterdam The Netherlands Institute of Medical Education Research Rotterdam, Erasmus MC University Medical Centre Rotterdam, Rotterdam, The Netherlands.; 2 Universidade de São Paulo Bauru SP Brazil Universidade de São Paulo, Bauru, SP, Brazil.; 3 Faculdade Pernambucana de Saúde Recife PE Brazil Faculdade Pernambucana de Saúde, Recife, PE, Brazil.; 4 Hospital Israelita Albert Einstein São Paulo SP Brazil Hospital Israelita Albert Einstein, São Paulo, SP, Brazil.

Moving towards an evidence-based education brings several challenges, including the underpinning foundations of current training. Simulation-based education (SBE) has been based on several rigorous research studies to demonstrate its efficacy. However, educators and researchers still use theories that are currently considered educational myths instead of theories with solid scientific evidence. In this editorial, we will describe several myths used in education and present theories that have been widely evidenced in the educational context. We hope this editorial will increase awareness of educators about educational myths and that they start using evidence-based theory in education.

## EDUCATIONAL MYTHS

The first myth we often encounter is about Edgar Dale's pyramid of learning^(1)^ which supposedly demonstrates the effectiveness of different forms of teaching. This myth lacks logic and evidence. We start with a simple question: how are you going to learn more by teaching without knowing the content? This simple question should suffice to prove it is a myth. As Master^(1)^ described nicely "Given that there is no research on which to base the Pyramid, and that Edgar Dale did not ever produce anything resembling the Pyramid, it is safe to say that the Pyramid and its associated percentages are entirely mythical." The continued use of this myth, besides being counterproductive, is also dangerous. Many educators use this myth to justify the use of simulation and to demonstrate that it leads to better educational outcomes. To take full advantage of SBE, learners need to possess the required competence for that specific training. If learners do not have the required competence, the SBE will be useless or at least provide limited educational gains. Also, we would like to make it a clear distinction: SBE does lead to increases in educational outcome and patient care^(2,3)^ However, this is not because of the myth of the Learning Pyramid.

The second myth is learning styles, which refers to the idea that people have different ways or preferences of learning and that the content should be adjusted to that. For example, if someone is a visual learner, then receiving the content in a visual format, such as videos and diagrams, will lead to better learning for that person. In contrast, an auditory learner would benefit from lectures and audio recordings. These seem logical to many people, as they often refer to themselves as one specific type of learner. However, learning styles are not supported by evidence. Well-designed and conducted studies demonstrate that learning is not related to learner styles (or preferences, since it is often self-reported), and often can hinder learning.^(4,5)^

The last myth is andragogy, a term that was first defined by Knowles. Knowles argued that adult learning differs from children's, citing a series of concepts, including motivation, past experiences, and meeting their needs.^(6)^ However, research from different fields of education and psychology, such as cognitive science, self-regulated learning, self-efficacy and others, demonstrates that the important concepts for learning are similar from children to adults. For example, both children and adults who have higher self-efficacy and self-regulation skills outperform those who have lower. From a memory perspective, learning is also the interplay between declarative and procedural knowledge, and the only difference is that adults possess a much larger knowledge structure than kids.^(7,8)^

So, the question becomes how people learn and how SBE fits in this context? Now, we will shortly describe the theories with most evidence and why they are important for SBE.

## EVIDENCE-BASED EDUCATIONAL THEORIES

Learning happens both from an individual perspective, by forming memories and beliefs, and is also socially constructed with others and environment. Bandura describes the concept of reciprocal determinism in which three elements; personal factors, behavior and environment, influence each other. The strength of each element will be different in each situation, but the three elements will always influence each other.^(9,10)^

Cognitive science offers insight into the memory system by dividing declarative and procedural knowledge. Declarative knowledge refers to facts and events, and everything we can remember. Procedural knowledge refers to the automatization of our processes, including declarative knowledge and motor actions. Learning will always start with declarative knowledge and with repeated practice will be developed into procedural knowledge. Noteworthy, declarative knowledge can decay over time, whereas procedural knowledge cannot. Expertise in this case is when knowledge is in procedural format, decreasing the time of execution and errors.^(11,12)^

Also, learners need to believe that they are capable of performing such tasks, known as self-efficacy. Self-efficacy beliefs have been one of the most predictive beliefs of performance. Learners who believe they are capable engage longer and are more resilient during learning, whereas learners who feel less capable may give up on learning quickly. Self-efficacy has been demonstrated to predict performance in different fields, such as mathematics, music and sports.^(13)^

Finally, the concept of self-regulated learning (SRL) is crucial as a metacognitive process in which learners actively manage their own thoughts, behaviors, and motivation.^(14)^ Although there are several models of SRL, most of them are cyclical and share at least three phases, before, during and after the task. Before the task, learners need to plan the strategies they will use, define goals and their beliefs, especially self-efficacy.^(15)^ During the task, learners need to monitor their performance and make adjustments. After the task, learners reflect on their performance and make a plan for how they will improve in future tasks. Studies demonstrate that learners with higher skills in SRL perform better than those with lower SRL skills. Also, SRL skills are stable across time and related skills.^(16)^

## IMPROVING SIMULATION-BASED EDUCATION

These theories also provide several strategies for how to increase learning and performance. These strategies can be combined or used individually, but we will provide a description of these strategies individually.

From a memory perspective, it is crucial to provide repeated and spaced training to learners, especially those who are learning new skills. Spaced practice has been shown to improve surgical skills^(17)^ resuscitation skills^(18)^ and others.^(19)^ Instead of providing a new opportunity for practice, learners could also be tested. Testing in this context is not about a formal assessment, but strategies to force learners to retrieve information from their memory without any aid.^(20)^ Educators should also interleave the practice with similar skills. Interleaving has limited evidence in medical education but has been shown to improve retention in cognitive psychology.^(21)^ All these strategies also help learners and educators avoid creating an illusion of knowing,^(22)^ which leads to the belief that learners acquired the trained skills, but when necessary they cannot perform adequately.

Feedback is one of the most important tools for educators.^(23)^ Educators often focus on the technical aspects of the task without considering the retention of those skills. There are two important aspects of feedback that are overlooked. First, the type of feedback given will influence the retention of the skills. Cecilio-Fernandes et al. compared three different types of feedback, one provided by an expert, another provided by the simulator, and a combination of expert and simulator feedback. During the training phase, learners who received expert feedback were much quicker in achieving the proficiency level. However, in the retention test, learners who received the combined feedback had better performance than the other two groups.^(24)^ Secondly, while feedback focuses mostly on the technical aspects of the task, feedback on SRL is crucial to develop learners’ metacognitive skills. Studies demonstrated that giving feedback on SRL also leads to improvement of skills. A particular method called microanalysis has been used to identify and give feedback in SRL skills, including self-efficacy. This method consists of structured questions about the SRL processes and can be tailored to each training. Providing feedback on self-efficacy will change learners’ beliefs about themselves, leading to higher engagement in the training.^(25)^

Research has been at the forefront of educational innovation and development of evidence. However, educators have often ignored evidence and trust more in their experiences. This shift from experience to evidence-based education brings a challenge to many educators, since much evidence may go against their own beliefs and experiences. Another challenge is that many of the educators responsible for teaching a specific content or skill do not necessarily follow up after a few weeks, when they could observe that learners are not able to apply their acquired knowledge and skills. A simple example is the training of basic and advanced support life, which mostly happens in sessions of one or two days. During the training, learners acquire a huge amount of content and skills, but after a few weeks there is a great decay in skills and knowledge. So, educators who gave the course observe and believe that learners acquired the necessary knowledge and skills, and learners also believe that since they were able to complete all the necessary requirements. After a period of time, the same learner cannot perform, and the previous educators are not present.

We hope that this editorial will guide educators to choose better teaching strategies and theory to underpin their simulation training. Also, that the myths used will disappear and we can undo many of the harms they caused.

## Data Availability

The content is already available.
